# Supplementary material for: Principal Component Analysis from Mass Spectrometry Data Combined to a Sensory Evaluation as a Suitable Method for Assessing Bitterness of Enzymatic Hydrolysates Produced from Micellar Casein Proteins
Source: Foods. 2020 Sep 24;9(10):1354. doi: 10.3390/foods9101354 (PMC7598618; doi:10.3390/foods9101354)
Supplement: Supplementary file 1 [file foods-09-01354-s001.pdf]

Supplemental data 1

In order to evaluate the performance and the reliability of the sensory panel, a three-way ANOVA (product, assessor, repetition) was done. In the following table, we have the associated probability (P) to each factor, as well as those of the two most interesting interactions between the factors (*e.g* product x assessor and product x repetition) to estimate the repeatability and the consensus between assessors.

Supplemental table 1 : panel performance details.

	ODOR					FLAVORS											PERSISTENCE OF FLAVORS				
**Descriptors**	**milk**	**fermented milk**	**rancid**	**soya milk**	**smelly**	**bitter**	**acid**	**milk**	**mild**	**sweet**	**rancid**	**whey**	**stable**	**cheese**	**vanilla**	**salted**	**bitter**	**sour**	**milk**	**sweet**	**cheese**
**P product**	0.00	0.02	0.00	0.07	0.00	0.00	0.00	0.00	0.00	0.52	0.00	0.27	0.08	0.00	0.03	0.05	0.00	0.00	0.15	0.76	0.00
**P repetition**	0.03	0.05	0.01	0.13	0.47	0.02	0.09	0.01	0.25	0.01	0.75	0.16	0.03	0.00	0.02	0.00	0.14	0.47	0.01	0.00	0.00
**P assessor**	0.00	0.00	0.00	0.00	0.00	0.00	0.00	0.00	0.00	0.00	0.00	0.00	0.00	0.00	0.00	0.00	0.00	0.00	0.00	0.00	0.00
**P product x assessor**	0.00	0.11	0.54	0.45	0.61	0.00	0.00	0.03	0.00	0.37	0.00	0.00	0.32	0.00	0.00	0.06	0.00	0.00	0.10	0.10	0.00
**P product x repetition**	0.31	0.56	0.88	0.43	0.08	0.71	0.23	0.71	0.18	0.65	0.67	0.89	0.63	0.66	0.19	0.27	0.26	0.02	0.88	0.75	0.85

The probabilities associated to the product factor show that the panel is quite discriminant since 14 descriptors out of 21 are well discriminated ($P<0.05$). Only the descriptors *soya milk odor, sweet, whey, stable, salted, milk and sweet persistence* are not discriminated.

Concerning the consensus, the interaction product x assessor is significant for 12 descriptors out of 21. However, a principal component analysis where the assessors are taken as variables and the products as observations, shows that the assessors are in agreement and that the difference observed was due to a scaling effect.

Regarding the panel repeatability, the interaction product x repetition shows that the panel is repeatable for all descriptors except for the descriptors *rancid odor* and *acid persistence*. The analysis of these two descriptors should be carried out with precaution.
